# Primary Tuberculous Meningitis

**Published:** 1906-02-17

**Authors:** 


### PRIMARY TUBERCULOUS MENINGITIS.

IT is an arguable question whether a primary infection of a serous membrane is an occurrence within the bounds of possibility or not: whether, that is to say, it is conceivable that an infecting agent can gain entrance to the blood-stream or directly to a serous membrane in sufficient quantity to produce a lesion without having first established itself in some other part of the body. On the one hand there is no doubt that cases are met with which appear to demonstrate such a direct affection without any intermediate base; while on the other it is a matter of great academic difficulty to conceive of an infecting organism passing not only through a mucous surface without causing a local lesion—for this is undeniably possible—but in addition escaping all the defensive mechanisms supplied by the lymphatic glands. The question is raised by the narration by Dr. H. W. Cheney [1] of a case of primary tuberculous meningitis. The case was not in itself an unusual one. A child of three and a half, while apparently in good health, was seized with vomiting. This persisted, and gradually the fatal significance of the symptom declared itself. The patient died on the twenty-fourth day of the illness. Post-mortem there was no evidence of any old lesion of tuberculosis which might be regarded as having served the capacity of a base for the organism. Now it must be readily recognised that, clinically speaking, primary tuberculous meningitis is a comparatively common disease, and one distressingly deceptive in its early stages; but post-mortem examination will almost certainly reveal an old focus of caseation though the patient may have been to all appearances in the most perfect health. This focus is, in the case of children—who supply almost all the instances of the disease—a glandular one. Of all sites commonly showing evidence of previously unsuspected tuberculosis the lymphatic glands situated at the bifurcation of the trachea take the first place in the matter of frequency, and pathological evidence points to these glands more often than to any other single tissue as the starting point of "primary" tuberculous meningitis. Second to the bronchial glands come the lymphatic glands of the mesentery, and in either case it is quite common to fail to find in the tissues served by these glands any evidence of tuberculous lesion. This very fact adds to the unlikelihood attending the presumed passage of tubercle bacilli through a series of lymphatic glands without arrest and local lesion. Dr. Cheney, by omitting any statement of the condition of the bronchial glands, has omitted a piece of evidence of the first importance, and one the lack of which robs his case of particular interest as a record of a rarity.

[1] Pediatrics, Oct., 1905.

